# Increases in Perspective Embedding Increase Reading Time Even with Typical Text Presentation: Implications for the Reading of Literature

**DOI:** 10.3389/fpsyg.2015.01778

**Published:** 2015-11-24

**Authors:** D. H. Whalen, Lisa Zunshine, Michael Holquist

**Affiliations:** ^1^Program in Speech-Language-Hearing Sciences, City University of New YorkNew York, NY, USA; ^2^Haskins Laboratories, New HavenCT, USA; ^3^Department of Linguistics, Yale University, New HavenCT, USA; ^4^Department of English, University of Kentucky, LexingtonKY, USA; ^5^Yale University, New HavenCT, USA

**Keywords:** reading, perspective embedment, literature, theory of mind, fiction

## Abstract

Reading fiction is a major component of intellectual life, yet it has proven difficult to study experimentally. One aspect of literature that has recently come to light is perspective embedding (“she thought I left” embedding her perspective on “I left”), which seems to be a defining feature of fiction. Previous work (Whalen et al., 2012) has shown that increasing levels of embedment affects the time that it takes readers to read and understand short vignettes in a moving window paradigm. With increasing levels of embedment from 1 to 5, reading times in a moving window paradigm rose almost linearly. However, level 0 was as slow as 3–4. Accuracy on probe questions was relatively constant until dropping at the fifth level. Here, we assessed this effect in a more ecologically valid (“typical”) reading paradigm, in which the entire vignette was visible at once, either for as long as desired (Experiment 1) or a fixed time (Experiment 2). In Experiment 1, reading times followed a pattern similar to that of the previous experiment, with some differences in absolute speed. Accuracy matched previous results: fairly consistent accuracy until a decline at level 5, indicating that both presentation methods allowed understanding. In Experiment 2, accuracy was somewhat reduced, perhaps because participants were less successful at allocating their attention than they were during the earlier experiment; however, the pattern was the same. It seems that literature does not, on average, use easiest reading level but rather uses a middle ground that challenges the reader, but not too much.

## Introduction

The reading of literature has received less attention in psychology than its ubiquity in culture would lead us to expect (e.g., Mar and Oatley, 2008; Popova, 2014). Why we read fiction has been a long-standing question with little consensus (e.g., Roche, 2008; Jusdanis, 2010). Whether it is for entertainment, enlightenment, or educational requirement, the consumption of stories in a wide range of genres remains a hallmark of human existence. One cognitive aspect of reading fiction that has been studied by Zunshine (2006) is the embedding of perspective. A statement such as “I went to the store” is a (putative) fact, but “Alice knows I went to the store” embeds that fact into Alice’s perspective. Such “embedment” (Dunbar, 2000) is critical to understanding a great deal of language, and much of literature (Zunshine, 2006, 2014). Zunshine’s (2014) estimation is that most literature communicates ideas, observations and information at a third level of embedment For example, in “She felt she knew too much about Rudi to respect him” (Spark, 1963, p. 59); the character has a mental representation of her attitude toward Rudi (i.e., she doesn’t respect him), and this state is embedded in her own knowledge of it, which is then reflected in her current feeling. Sometimes literature reaches the fifth or sixth level. The ability to discuss perspectives of those not present opens the possibility of considering the perspective of “people” who were never present, that is, fictional characters.

Cognitive science, like linguistics, has often been concerned primarily with the power of language to transfer and structure information. Language is often taken as being primarily a cognitive tool, a “system for expression of thought” (Chomsky, 1975, p. 57). Similarly, the organization and retrieval of information is a central concern of psychology. The claimed preference for embedment in literature, then, seems on the surface to be contrary to our cognitive core. Although facts are things that we typically care about and seem to be a major factor in our linguistic structure, humans are social animals intent on communication (e.g., Butterworth, 1994), which often depends on the state of knowledge both of the speaker and the interlocutor, that is, embedding of perspective.

There are many linguistic devices for conveying levels of embedment. The ones used in the previous examples are the most obvious, the “mental state” verbs (Zunshine, 2014). Some languages, such as Shipibo-Konibo (Valenzuela, 2003) or Salar (Dwyer, 2000), require a sentence to be marked grammatically for the speaker’s engagement with the facts. The suffixes or other morphemes encoding this information are called “evidentials” and often mark whether a speaker knows the fact from personal experience, has heard about it, or if it is a universally accepted truth (Chafe and Nichols, 1986; Aikhenvald, 2004). Languages that lack grammatical evidentials usually have periphrastic ways of expressing the same meanings (“I see that you have a cough” (direct evidence) vs. “I understand that you have a cough” (reported evidence) vs. “One knows that coughs are dangerous” (universal truth). Such devices signal an initial estimate of the embedment level of a sentence, but they are not the only means available to speakers and writers. Implied embedments can be suggested by a variety of stylistic techniques, ranging from the use of metaphor, aphorism, and allusion, to the strategic stripping of the text from any references to mental states (Zunshine, 2011; Bowes and Katz, 2015). For example, “I drove the motorcycle half-way across the country” implies a perspective from which the journey can be seen as only half-way and not simply a certain number of miles. Such stylistic devices tend to accumulate in a text, as discussed more fully in the cited articles.

Despite similarities, perspective embedment is not the same as syntactic embedding or visual perspective-taking. Syntactic embedding, a hallmark of human language (Chomsky, 1965), can occur without an increase in perspective embedding. Thus “she thought that he had left” embeds in both ways, while “she saw the dog that bit the man” only embeds syntactically. It is quite possible that perspective embedding usually requires syntactic embedding, but the reverse is certainly not true. In the same vein, participants have been shown to take the visual perspective of a participant in a scene when they place “good” and “bad” objects in a visual scene (e.g., Kominsky and Casasanto, 2013), but such an operation instead takes the original observers perspective *out* of the situation rather than embedding it. That is, it is not the participant’s perspective on the actor shown in the picture (embedment), but rather the participant imagining seeing the world from the actor’s perspective (a change rather than an embedment). It is essentially an exchange rather than an embedment.

Perspective embedment is essential for engaging readers in fiction. (By fiction we mean novels, short stories, drama, narrative poems, and memoirs focused on imagination and consciousness.) Readers of fiction (typically) know that they are reading about events and feelings that did not happen in the real world, and yet they can become deeply immersed in those stories. The ability of language to draw us into such stories is probably unique to human language (Hockett, 1960) and appears early in acquisition. By preschool, children show the capacity to contemplate embedded mental states; the ability correlates positively with rich vocabulary and practices of story-reading, and this correlation becomes even stronger as children grow older (Peskin and Astington, 2004; Harris, 2005).

Fictional embedment of mental states is arguably related to our tendency to construct complex embedments in our daily social life (Zunshine, 2015), yet one can argue that popular literature makes use of more overt indicators than does literature in the canon. Works of fiction taught in literature classes make their readers work harder at figuring out embedded mental states (Zunshine, 2014). The perceptual processes and cognitive tasks that occur during reading (both of literature and other texts) are numerous (e.g., Pugh et al., 1996; Rayner et al., 2001; Long et al., 2006; McNamara and Magliano, 2009; Forrest-Pressley and Waller, 2013). There are always aspects of reading that correlate, even if they are dissociable at some level. The present experiments will not be able to determine the exact level of cognitive assessment of our texts. Instead, they present responses to texts that have been designed to vary a dimension, perspective embedment, that has not been addressed by others. Only future work will be able to define the cognitive processes involved more completely, but we hypothesize that constructing interpretations of embedment is one more process that can be identified. The mechanics of that construction remain to be determined. As an ancillary, we explore whether having only one actor in a vignette versus having three impacts reading time and, by inference, processing time.

In our previous study of embedment (Whalen et al., 2012), we used reading time as an index of processing difficulty (cf. Rayner et al., 2006; Feng et al., 2013). We constructed vignettes of a consistent length that varied in the level of embedment. Those with no mental states were at level 0, while adding one or more mental state allowed us to increase the embedment from 1 to 5, and, by assumption, increased complexity. Every sentence in every vignette was judged by us to be at the stated embedment level. These judgments have been found to be largely replicable (Whalen et al., submitted-b). A further manipulation was to have only one actor in the vignette (the narrator “I”) or three. If the mental states of a single individual were not challenging in the same way as mental states for a variety of actors, then increasing embedment for the single actor vignettes might not increase in complexity in the same way as the three actor ones. We found that vignettes with embedment levels ranging from 1 to 5 increased reading time almost linearly, while level 0 was between 3 and 4 in reading time. The pattern was basically the same for the single actor and the three actor vignettes, although the single actor ones were consistently somewhat faster. We concluded that mental states do increase the mental processing that must go on during reading, even though we were unable to determine the exact nature of those processes from those two experiments.

In the previous study, a moving-window paradigm (e.g., Just et al., 1982) was used to ensure that regressions (i.e., re-reading text) would not occur, given that regressions, though natural, greatly affect overall reading time and are themselves more common in difficult texts (e.g., Rayner and Well, 1996; Rayner et al., 2006). However, that paradigm also introduces a level of unnaturalness in the reading process that might interact with the effects of embedments. To explore this possibility, the present experiments replicated the earlier study with full presentation of the vignettes (that is, “typical” presentation), either for a participant-determined amount of time (Experiment 1) or for a fixed amount of time (Experiment 2).

## Experiment 1

In Whalen et al. (2012), vignettes of a consistent length were presented in the moving-window paradigm to allow us to obtain reading times with no regressions. Although eliminating regressions, this demanded of participants an unpracticed form of reading, which may have led to unanticipated strategies for comprehension. The present study was designed to avoid that possibility by allowing the participants to view the entire vignette at once, as is the case with most reading situations. We then compared their reading times and accuracy on probe questions to the results from the earlier study. We expected the more natural reading situation to lead to longer reading times (presumably including regressions) and somewhat more accurate responses, although many of the previous accuracy levels were near ceiling.

### Methods

#### Participants

The participants were 16 college educated young adults (10 female) with no reported speech or language pathologies. They were paid for their participation. All signed a consent form approved by the City University of New York Institutional Review Board.

#### Stimuli

The 84 vignettes of Whalen et al. (2012) were used (available at https://yale.box.com/s/qvk12d3vwrppimedrrdkkj5hgrdq5p76). These were designed to have embedment levels ranging from 0 to 5; the reliability of these designations has been established earlier (Whalen et al., submitted-b). There were either three actors or one (the narrator, “I”); the narrator was often an actor in the three actor vignettes. The addition of explicit characters allowed us to examine whether the “virtual” actor in the one-actor vignettes took as much time to encode as the genuine additional actors. There were seven vignettes at each of these 12 combinations of actors and embedments. Every sentence was designed to be at the target embedment level. We ensured that the number of text characters (including the alphabetic ones and punctuation, but excluding spaces) in each vignette was 350. They averaged 426.1 characters with spaces (ranging from 412 to 436). The number of lines presented was always 7. This facilitated the earlier study’s use of the moving window technique. The last line was always about half as long as the other lines. The number of punctuation marks averaged 6.2 (ranging from 5 to 9). The number of sentences averaged 4.6 (ranging from 2 to 10) while the number of words per sentence averaged 20.2 (ranging by vignette from 9.4 to 42.0). The number of words per vignette averaged 84.4 (ranging from 69 to 95).

Each vignette was followed by a probe question. To ensure a full reading of the vignette, that question was typically designed to require analysis at the highest level of embedment. This was to prevent a strategy of reading quickly for the most superficial facts. For the higher levels of embedment, it was difficult to construct a short question that was dependent on the embedment and yet with a clear “yes/no” answer. Thus some of the “incorrect” answers at higher levels are, in fact, open to legitimate disagreements. Nonetheless, to be consistent with the previous study, the designated correct answer is taken as the correct answer here. Note that the high level of agreement on levels 0–4 was taken as validation of our assignment of correctness of the probe questions.

#### Procedure

Vignettes were presented as typical text, that is, with all words presented at once rather than one segment at a time as had been done in the previous, moving-window experiments. The text appeared on a computer screen under the control of the computer program Presentation^®^ (www.neurobs.com). The participants were instructed to read each vignette for as long as they thought they needed to in order to be able to answer a yes/no question about the content. Once they had finished reading, they pressed a key, and the probe question replaced the vignette on the screen. The probe question remained available for 7 s. No time pressure was stated for this judgment (other than the 7 s limit). A different order of vignettes was used for each participant, and each responded to every vignette.

### Results

#### Reading Time

All reading times for all the vignettes were analyzed regardless of the correctness of the answer. This was the technique used in the previous study, based on the fact that level 5 had disproportionate numbers of errors. Visual inspection of means with and without errors showed no differences in the pattern of results. It can be assumed that errors did not affect the reading time of the vignette being probed, and the lack of time pressure (and feedback) meant that the upcoming reading time was unlikely to be affected either.

A linear mixed effects model (LME) was performed using the lmer function from the lme4 package (Bates et al., 2013) in R, version 3.2.2 (R Core Team, 2015). An analysis with random slopes and intercepts failed to converge, so the simpler analysis with intercepts only was performed, as recommended by Barr et al. (2013). This analysis had the fixed the factors Embed (ordered nominal levels 0–5) and Actors (1 or 3 actors); the interactions were tested as well. Reading time was the dependent variable with crossed random intercepts for participant and item. Results are shown in **Table 1**.

**Table 1:** Linear mixed effects model (LME) of reading times for Experiment 1.

	Estimate	Standard error	*t* value	Confidence interval	
(Intercept)	21471.0	1784.8	12.030	17941.6	25000.3
Embed 1	-3355.8	1061.5	-3.161	-5382.7	-1328.9
Embed 2	-1844.3	1061.5	-1.737	-3871.2	182.5715
Embed 3	-705.1	1061.5	-0.664	-27312.0	1321.8
Embed 4	1965.7	1061.5	1.852	-61.2	3992.6
Embed 5	3708.0	1061.5	3.493	1681.1	5734.9
Three actors (v. 1)	3902.8	612.9	6.368	2732.6	5073.0
Embed 1 ^∗^ 3 Actors	-866.9	2045.3	-0.424	-4643.4	2909.7
Embed 2 ^∗^ 3 Actors	887.8	2045.3	0.434	-2888.7	4664.4
Embed 3 ^∗^ 3 Actors	113.3	2045.3	-0.055	-3889.9	3663.2
Embed 4 ^∗^ 3 Actors	2112.0	2045.3	1.033	-1664.5	5888.6
Embed 5 ^∗^ 3 Actors	5017.7	2045.3	2.453	1241.1	8794.2

**FIGURE 1:**
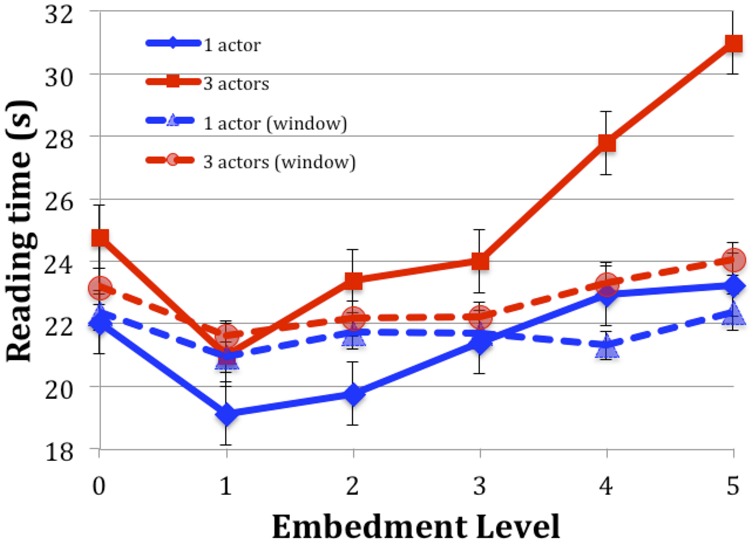
**Comparison of current results (solid lines) with those of Experiment 1 of Whalen et al. (2012) (dashed lines, “window”).** Reading times are averaged across participant and vignette. Error bars represent one SEM.

The reading times showed essentially the same pattern as in the previous results (**Figure 1**), although overall times were longer (by an average of 1.13 s), substantially so at the fifth level (7.82 s).

A second LME analysis compared the results with those of Whalen et al. (2012) by adding the fixed effect Experiment [current (full) vs. previous (window)].

Interactions tested were Embed by Experiment and Actors by Experiment. The three-way interaction did not converge. Because the resulting table is quite large and mostly non-significant, only those effects with confidence intervals that do not include zero are reported here. Reading times for Embedment level 1 were shorter in the current experiment (*t* = -0.217, CI = -3558.7/-184.3). For Embedment levels 4 and 5, times were longer in the current experiment (*t* = 2.82 and 3.79 respectively, CI = 740.6/4115.1 and 1578.2/4952.6). The three Actor vignettes took longer to read in the present experiment (*t* = 5.78, CI = 1897.0/3845.2).

#### Accuracy

**FIGURE 2:**
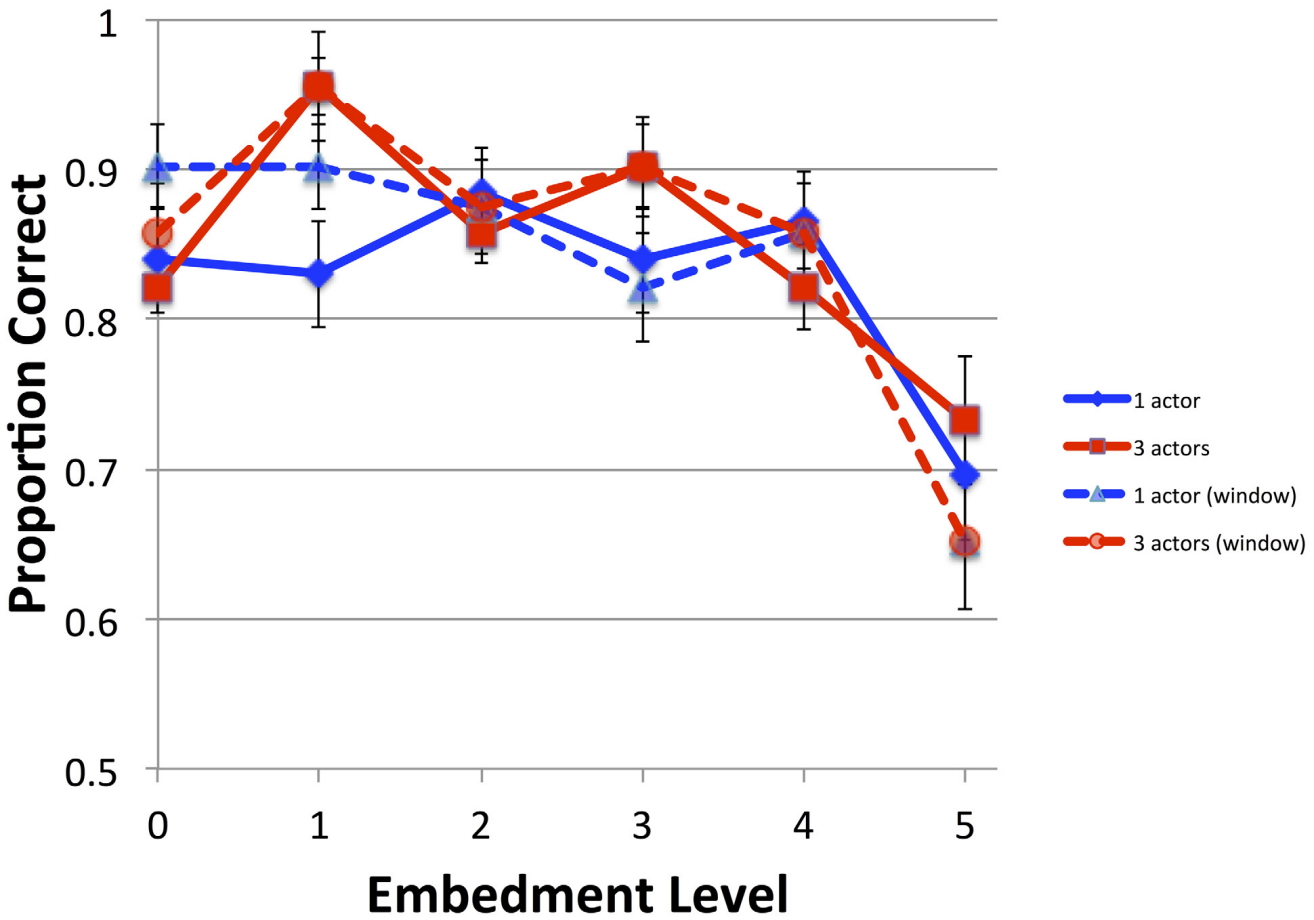
**Accuracy for Experiment 1 (solid lines) compared with those of Experiment 1 of Whalen et al. (2012) (dashed lines, “window”).** Error bars are one SEM.

Accuracy on the probe questions was quite good except for the level 5 and, for the three actor vignettes, the level 4 (see **Figure 2**). The present results showed a similar pattern to previous results, as seen in **Figure 2**.

A generalized linear mixed model with logistic regression (GLM; glmer from the lme4 package in R) was performed with the factors Embed (levels 0–5), Actors (1 or 3 actors) and crossed random intercepts for participant and item. Response accuracy (0 or 1) was the dependent variable. **Table 2A** presents the results for Experiment 1, with comparisons made to Embedment Level 5 rather than 0, as the previous experiment had shown that level to be the one that was different from the others. It was found to be different from levels 1, 2, and 3, but not 0 and 4. The number of actors did not affect the accuracy.

**Table 2:** GLMM of percent correct: (A) Experiment 1, (B) Comparing previous experiment.

	Estimate	Standard error	*z* value	Pr(>|z|)
**(A)**				
Embed 4	0.858	0.470	1.826	0.068
Embed 3	1.189	0.479	2.484	0.013^∗^
Embed 2	1.100	0.477	2.304	0.021^∗^
Embed 1	1.314	0.486	2.704	0.006^∗∗^
Embed 0	0.774	0.468	1.652	0.098
Three Actor	0.206	0.283	0.729	0.466
**(B)**				
Embed 4	1.233	0.460	2.680	0.007^∗∗^
Embed 3	1.295	0.461	2.808	0.005^∗∗^
Embed 2	1.573	0.472	3.335	0.0009^∗∗∗^
Embed 1	2.146	0.496	4.329	1.5e-05^∗∗∗^
Embed 0	1.628	0.474	3.435	0.0006^∗∗∗^
Three actor	0.169	0.25429	0.663	0.507
Experiment	0.370	0.257	1.443	0.149
Embed 4 ^∗^ Experiment	-0.409	0.357	-1.145	0.252
Embed 3 ^∗^ Experiment	-0.098	0.372	-0.262	0.793
Embed 2 ^∗^ Experiment	-0.41	0.374	-1.111	0.266
Embed 1 ^∗^ Experiment	-0.855	0.410	-2.083	0.037^∗^
Embed 0 ^∗^ Experiment	-0.845	0.369	-2.290	0.022^∗^

*^∗^p < 0.05; ^∗∗^p < 0.01; ^∗∗∗^p < 0.001.*

A second analysis compared the current results with those from the previous study by adding the fixed effect Experiment [window (previous) vs. full (current)]. Models with interactions that included Actors did not converge, so only the interaction between Experiment and Embedment was analyzed. As **Table 2B** shows, all the Embedment levels were distinct from 5 on this analysis, and there were interactions between Experiment and levels 0 and 1.

### Discussion

Readers took longer to read vignettes as the embedment level increased from 1 to 5. Level 0, however, was between 3 and 4. The added complexity of multiple perspectives seems to have led readers to spend more time understanding the texts. Accuracy on the probe questions only dropped off at level 5, which also included some questions with arguably ambiguous answers. The longer times at level 0 could either be due to a lack of engagement on the part of the reader, or perhaps to a greater density of factual information. Current measures of information density do not provide any direct answer to this possible difference. Vignettes with three actors elicited longer reading times than those with one actor, a further indication of the effects of complexity of the text on reading time. For either number of actors, the steady increases in reading time due to increasing embedment indicate that perspective requires some processing even when only one person is involved. That is, in the one actor vignettes, the narrator’s perspective on the story acts is equivalent to a “virtual person” as far as reading time is concerned.

The presentation of text in the current experiment was much closer to the way reading is typically performed, yet the results were similar to those obtained with the moving window technique (Whalen et al., 2012). Although the reading times were, on average, longer than in the moving-window experiment (23.4 vs. 22.3 s), they did not differ significantly, even though the full presentation was more practiced than the moving-window version. We do not know how many regressions occurred during the reading, because we did not use an eye-tracker. However, regressions are normal and would be expected to rise as difficulty rises, consequently increasing reading time. Indeed, reading times in this study were sometimes faster than in the earlier study (for level 1). So the lack of pressure on reading time and presentation method allowed for even more attention to the level 5 vignettes, particularly for the three actor ones. This further confirms that the difficulty we inferred before is present even in a more normal reading style.

There was virtually no difference in accuracy between the present results and the earlier ones. Any null effect must be treated with caution, of course, especially with relatively small sample sizes. Nonetheless, individual points along the continuum differed a bit, but the overall pattern was the same: only the difficult questions at level 5 caused a significant decline in accuracy. There was no obvious benefit to using a typical reading paradigm rather than the moving-window presentation as in the earlier study. This decline occurred despite (or perhaps because of) the additional time taken in reading the level 5 vignettes in the current study. As mentioned, the construction of the probe questions for level 5 was difficult because we needed short yes/no questions that occasionally depended on understanding the higher embedment *per se*. Some of the answers could have been argued with; it may be that these disagreements were given greater weight with the increased time to encode the vignettes. In any event, it is clear that the overall pattern was the same in both experiments, further supporting the idea that the probe questions were successful at ensuring that the vignettes were read fully and that there was no speed-accuracy trade-off.

## Experiment 2

The second experiment mirrored the second experiment of Whalen et al. (2012) in that the vignette was always visible for 23 s, no matter what its embedment level. That duration had been chosen to prepare for a functional magnetic resonance imaging (fMRI) study (Whalen et al., submitted-a). In the moving-window paradigm, the participants could tell when the vignette was about to end. With the full-paragraph presentation used here, they could not. Therefore, we added a visible countdown of the total number of seconds so that any last second (re)readings could be performed. Despite the results of Experiment 1, it was still our prediction that the typical reading style available to the participants in this experiment (in contrast to the earlier study) would result in more accurate responses.

### Methods

#### Participants

The participants were 12 college-educated young adults (six female) with no reported speech or language pathologies. Three had acquired English early (before 7 years of age) but not first; all considered English their primary language. They were paid for their participation. None had participated in Experiment 1. All signed a consent form approved by the CUNY Institutional Review Board.

#### Stimuli

The stimuli were those of Experiment 1.

#### Procedure

Participants were instructed to read the vignettes for 23 s. There was a number in the lower left of the screen that counted down from 23 to 0, so that they could tell when the time was running out. They were told to answer the yes/no probe question that came afterward; it remained available for 7 s, as in the previous experiment. No time pressure was stated for this judgment (other than the 7 s limit). The next vignette appeared after a 2-s pause. There was an option to take a break after 42 vignettes.

### Results

**FIGURE 3:**
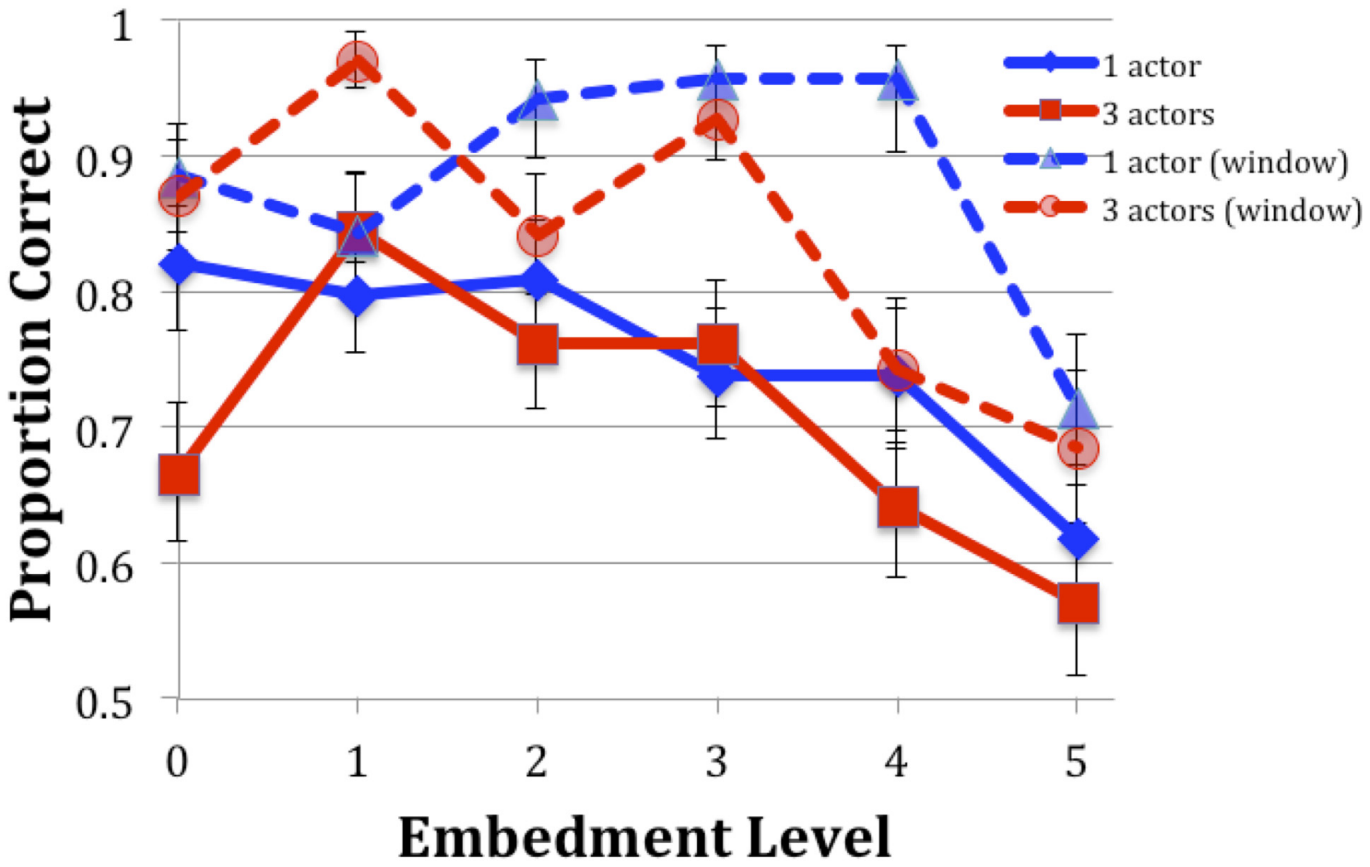
**Accuracy for Experiment 2 (solid lines) compared with those of Experiment 2 of Whalen et al. (2012) (dashed lines, “window”).** Error bars are one SEM.

Overall, responses were 73.1% accurate, treating both incorrect responses and the 1.5% missing responses as errors. This compares with 82.8% in Experiment 1. It is also lower than the 86.2% of Experiment 2 of Whalen et al. (2012). The pattern of errors, however, was very similar in those two experiments (see **Figure 3**).

A generalized linear mixed model with logistic regression (GLM; glmer from the lme4 package in R) was performed with the factors Embed (levels 0–5), Actors (1 or 3 actors), the interaction, and crossed random intercepts for participant and item. (An analysis with random slopes, as with the first experiment, did not converge.) Response accuracy (0 or 1) was the dependent variable. **Table 3A** presents the results for Experiment 2, with comparisons made to Embedment Level 5 rather than 0, as with Experiment 1. It was found to be different from levels 0, 1, and 2, but not 3 and 4. The number of actors did not affect the accuracy, and none of the interactions were significant.

**Table 3:** GLMM of accuracy data: (A) Experiment 2; (B) comparison with earlier study.

	Estimate	Standard error	*z* value	Pr(>| z|)
**(A)**				
Embed 4	0.675	0.536	1.26	0.209
Embed 3	0.724	0.542	1.34	0.182
Embed 2	1.127	0.550	2.05	0.040^∗^
Embed 1	1.090	0.551	1.98	0.048^∗^
Embed 0	1.253	0.557	2.25	0.024^∗^
Three actor	-0.237	0.521	-0.45	0.650
Embed 4 ^∗^ 3 Actor	-0.283	0.752	-0.377	0.706
Embed 3 ^∗^ 3 Actor	0.305	0.762	0.401	0.689
Embed 2 ^∗^ 3 Actor	-0.017	0.772	-0.022	0.983
Embed 1 ^∗^ 3 Actor	0.539	0.784	0.69	0.492
Embed 0 ^∗^ 3 Actor	-0.769	0.765	-1.01	0.314
**(B)**				
Embed 4	1.2188	0.510	2.39	0.017^∗^
Embed 3	2.3521	0.582	4.04	5.35e-05^∗∗∗^
Embed 2	1.5951	0.524	3.04	0.002^∗∗^
Embed 1	1.7624	0.534	3.30	0.001^∗∗∗^
Embed 0	1.3969	0.511	2.71	0.007^∗∗^
Three actor	-0.3712	0.261	-1.42	0.155
Experiment	-0.5360	0.379	-1.41	0.158
Embed 4 ^∗^ Experiment	-0.6407	0.421	-1.52	0.128
Embed 3 ^∗^ Experiment	-1.4256	0.504	-2.82	0.005^∗∗^
Embed 2 ^∗^ Experiment	-0.3828	0.440	-0.87	0.384
Embed 1 ^∗^ Experiment	-0.2804	0.455	-0.61	0.538
Embed 0 ^∗^ Experiment	-0.4941	0.426	-1.16	0.246

*^∗^p < 0.05; ^∗∗^p < 0.01; ^∗∗∗^p < 0.001.*

**Table 3B** shows the comparison with the previous study, as before. Every embedment level differed significantly from level 5. Experiment did not appear as a main effect. Only Embedment level 3 entered into an interaction with Experiment. Because many of the distributions of errors between the two studies were non-overlapping (**Figure 3**), a separate GLMM with only the factor Experiment was run. A *z* value of -3.25 (*p* = 0.001^∗∗^) was found. Given the small sample size and the lack of an effect in the larger analysis, this result should also be treated with caution. Overall, however, there is no evidence that the typical presentation improved performance over the moving window presentation of the earlier study.

### Discussion

The reading time available to participants was the same as in the second experiment of the previous study, but the experience seems not to have been the same. With the moving-window technique, the participants did not need to plan their reading strategy nor to keep track of the time, because they had no control over it; here, they needed to plan for the conclusion of the reading time. It is possible that the timer we used was not effective in signaling the stimulus’s approaching end, but that was not the typical report from participants; they said that the timer was annoying at first but routine by the end. It is more likely that the need to divide attention between reading and timing reduced the uptake of information. However, the overall pattern, with only level 5 being significantly worse, was the same in both experiments

The decline in accuracy from the window presentation to the full paragraph presentation demonstrates that an unusual presentation method for reading does not necessarily lead to less efficient reading. This decline could be due to (perhaps unnecessary) regressions; because participants expected to get more out of a vignette when they could see it in its entirety, or some other cause. In any event, the more natural condition of presenting the text in its entirety, rather than a segment at a time, led to worse performance. It seems unlikely that preventing regressions leads to better comprehension in general, but we do not know of any direct test of that possibility.

## Comparison with Discourse Measures

The focus of these experiments has been on perspective embedment, but, as noted in the Introduction, there are necessary correlations with other phenomena. Again, perspective embedment is different from syntactic embedding, but it typically requires more complex syntax, especially with the overt verbs of cognition used here. There are also many other factors that affect reading time, such as word frequency, that could not be controlled in the design of these vignettes. As a qualitative assessment, we compare the results from Experiment 1 to various metrics generated by Coh-Metrix (McNamara et al., 2014). Some of the comparisons are qualitative due to the short length of our texts [they averaged 84.4 words per vignette, while McNamara et al. (2014, pp. 153–154) recommend a minimum of 100], and the relatively small sample size of the present experiment. Some measures, such as average sentence length and word frequency, are not affected by the size of the text and can be correlated with embedment level. The Coh-Metrix index is given with the *r* value.

The number of sentences decreased with increasing embedment (DESSC, *r* = -0.733, *p* < 0.001; all dfs in this section are 82, means are in **Table 4**). Sentence length, therefore, increased with higher embedment (DESSL, *r* = 0.800, *p* < 0.001). The number of syllables per word increased slightly with embedment level (DESWLsy, *r* = 0.246, *p* < 0.05). Word frequency for content words as measured in the CELEX database (Baayen et al., 1995) was not deliberately controlled, but it turned out not to correlate with embedment level (WRDFRQc, *r* = 0.009, n.s.).

**Table 4:** Values of selected indices from McNamara et al. (2014), averaged across vignettes.

Embedment level	# Sentences per vignette	# Words per sentence	# Syllables per word	CELEX word frequency	Flesch Reading Ease	Flesch Reading Ease, one actor	Flesch Reading Ease, three actors	Flesch-Kincaid Grade Level
0	5.21	16.38	1.38	2.35	73.35	73.35	73.36	7.10
1	6.29	14.29	1.29	2.45	83.01	81.65	84.36	5.23
2	5.71	15.39	1.32	2.62	79.33	76.00	82.66	6.02
3	4.36	19.32	1.38	2.43	70.70	68.79	72.62	8.20
4	3.21	26.17	1.41	2.42	60.61	57.91	63.31	11.31
5	2.86	29.77	1.40	2.48	58.60	53.78	63.42	12.48

*N = 84 for most columns, 42 for the 1 and 3 actor measures of the Flesch Reading Ease columns. Coh-Metrix indices are: DESSC, number of sentences; DESSL, average sentence length; DESWLsy, average number of syllables per word; WRDFRQc, average CELEX word frequency for content words; RDFRE, Flesch Reading Ease; and RDFKGL, Flesch-Kincaid Grade Level.*

Portions of the pattern for several reading difficulty measures are consistent with the reading times in Experiment 1, but the differences are instructive. The Flesch Reading Ease measure (Flesch, 1948) and the Flesch-Kincaid Grade Level (Kincaid et al., 1975) were taken from the algorithm of McNamara et al. (2014). They mirrored the overall pattern for embedment, largely due to the influence of sentence length, which covaried to some extent with embedment but increased for the level 0 vignettes. However, the values for the three actors vignettes are measured as being more readable than those of the one actor at every embedment level; this is the opposite was found in the reading times. Further, the level 0 vignettes are placed between levels 2 and 3 in the reading measures, but between 3 and 4 in Experiment 1’s reading times.

We should not expect to be able to distinguish all of these factors with any one experimental stimulus set, much less one that is exploring a factor for the first time. The present results should provide a stimulus to further study to examine the effects of these new factors. We would expect that including some aspects of perspective embedment would lead to better measures of readability. As a first pass, treating verbs that usually signal embedment (think, know, believe) could be coded in addition to current factors.

## General Discussion

The present experiments show that adding multiple viewpoints on the information in a vignette—perspective embedment—increased reading time in a self-paced task for those vignettes with some embedment. In Experiment 1, for vignettes without embedment (“Level 0”), reading times were between those of levels 3 and 4. Accuracy on probe questions did not decline until level 5. It appears that the additional complexity of the multiple perspectives makes reading more difficult. Level 0 may be slow due to lack of engagement (i.e., readers expect stories to include human perspectives) or to an overall increase in the number of details in those vignettes; measurements of information density, which might distinguish these possibilities, do not currently provide an answer. In Experiment 2, with reading time fixed, accuracy dropped. It is likely that participants did not adopt a strategy that allowed for complete readings. The time allowed (23 s) replicated the times used in our earlier study (Whalen et al., 2012) for a moving window display. Because regressions were possible in this experiment, it may be that participants made use of them even though there was insufficient time to benefit from them. An eye-tracking study would help resolve this question. The pattern of errors, however, was similar to Experiment 1 and the previous study, with level 5 being the lowest. Overall, the present results indicate that a reader’s experience with a text is affected by the structure of the information given by the differing perspectives of the actors within that text.

The results from the present experiments were similar to those from the previous study despite their more natural reading conditions. Our expectation that natural reading conditions, including the one in which the participants could read the text for as long as they wished, would lead to greater accuracy on the probe questions was not met. Instead, there was a noticeable decrement in accuracy; the reason for this decline is unclear. It may have to do with the use of regressions in reading. Regressions are natural in reading and help the reader understand difficult or ambiguous material. Allowing regressions in Experiment 1, however, did not lead to improved accuracy over the previous study (where regressions were not allowed). In Experiment 2, where there was a time constraint, the time taken for the regressions may have given the reader a better understanding of the particular sentence that elicited the regression, but that may have left too little time for reading the rest of the sentences. Alternatively, it could be that preventing regressions improves reading understanding, perhaps by forcing greater attention as the information progresses. This has not, to our knowledge, been tested, but it would be similar to the decline in memory tasks when participants believed that they would be able to look up the information repeatedly on the internet (Sparrow et al., 2011). Further, longer reading times have been linked with greater acceptance of misinformation from fictional short stories (Fazio and Marsh, 2008), suggesting that extending the time allowed to process information is not always helpful. Only further work would clarify this.

The present experiments measured levels of embedment in sentences and short vignettes, so the question arises whether such stimuli adequately model what is going on in works of fiction. At the least, such judgments can be made on extracts from novels, as shown in Experiment 2 of Whalen et al., submitted-b. There, the first 12 sentences of “To Kill a Mockingbird” (Lee, 1960) were analyzed by the experimenters and by students of literature. For those, the agreement on embedment level was 49.3% (compared with 74.5% for the vignettes). The judgments are clearly less consistent for this segment of literature, eliciting less consistent judgments than in the present experiments and raising interesting questions about the reasons for the differences.

The vignettes under study here have also been presented to participants in an fMRI experiment (Whalen et al., submitted-a). Regions associated with Theory of Mind were more active for vignettes with embedments than for those lacking them altogether. Although those results are preliminary, they indicate that the process of reading fiction recruits brain regions arguably responsible for complex embedments that structure our social life (e.g., I didn’t want my boss to know what I was thinking). Works of fiction intensify such real-life embedments, foreground their emotional salience, and drastically increase the frequency of their appearance, but their underlying structure (i.e., mental states within other mental states) is preserved.

The experiments reported here and in our related publications indicate that readers are sensitive to different levels of embedment and that experimenters can be reasonably consistent in their assigning the levels. Given that describing the level of embedment in connected discourse is an important new way of interpreting the information structure of texts, evidence of such consistency is encouraging.

## Conflict of Interest Statement

The authors declare that the research was conducted in the absence of any commercial or financial relationships that could be construed as a potential conflict of interest.
